# The Effect of a Novel Achilles Brace on Concentric and Eccentric Achilles Tendon Loading During Tendon Tear Mechanisms

**DOI:** 10.3390/life16030524

**Published:** 2026-03-21

**Authors:** Roni Gottlieb, Shai Greenberg, Asaf Shalom, Julio Calleja Gonzalez

**Affiliations:** 1 School of Human Movement and Sport Sciences, The Levinsky-Wingate Academic College (Wingate Campus), Netanya 4290200, Israel; shai@plife.co.il; 2 Department of Physical Education, The Research Center for Sports and Physical Activity, Tel Hai Academic College, Kiryat Shmona 1220800, Israel; asaf.fitness@gmail.com; 3 Department of Physical Education and Sports, Faculty of Education and Sport, University of the Basque Country, UPV/EHU, 01007 Vitoria-Gasteiz, Spain

**Keywords:** ankle, concentric, eccentric, Achilles tendon, injury

## Abstract

(1) Achilles tendon rupture is one of the most severe lower-limb injuries, frequently occurring during movements involving maximal dorsiflexion with the knee at near-full extension. Preventive strategies are crucial, particularly for athletes engaged in high-risk sports such as basketball. (2) In this work, we examined the effect of a novel Achilles brace on Achilles tendon loading during concentric and eccentric mechanisms associated with tendon rupture. (3) Twenty-eight young basketball players performed tests under two conditions: with the adaptive brace and without it (control). Participants were divided into two groups (n = 14 in both). The first group assessed concentric Achilles loading by performing three plantar-flexor strength tests in three different joint configurations: maximal dorsiflexion with the knee flexed (FKF); injury mechanism position—full plantar flexion with the knee extended (FKE); and neutral ankle position with the knee extended (NKE). The number of maximal heel-raise repetitions performed before onset of fatigue was recorded. The second group assessed eccentric tendon loading by performing single-leg forced maximal-velocity dorsiflexion with the knee extended. In all tests, the time between maximal plantar flexion and maximal dorsiflexion, as well as the ankle range of motion, was analyzed using 2D video. Paired *t*-tests were used to compare braced and control conditions. In all tests, the ankle range of motion (ROM) did not differ significantly between brace and control conditions. Wearing the brace significantly improved plantar-flexor muscle strength only in the FKE test (31 ± 1.3 repetitions with brace vs. 21 ± 1.3 in control, *p* < 0.05). No significant differences were found for the FKF (27 ± 1.3 vs. 25 ± 1.3) or NKE (25 ± 1.3 vs. 24 ± 1.3) positions. During drop eccentric loading, wearing the brace resulted in a significantly slower transition time from plantar flexion to dorsiflexion (460 ± 60 ms with brace vs. 320 ± 30 ms in control, *p* < 0.001). (4) In brief, the novel Achilles brace was found to significantly reduces Achilles tendon load during both concentric and eccentric activities, but only in high-risk joint positions. These findings suggest that the brace provides mechanical protection, and may reduce the risk of Achilles tendon rupture, in athletes exposed to high tendon stress.

## 1. Introduction

The Achilles tendon is the strongest and thickest tendon in the human body; however, it remains highly susceptible to injury, including complete or partial ruptures [1], as well as chronic-overuse pathologies such as tendinosis [2]. This paradox is explained by the exceptionally high mechanical demands placed on the tendon during dynamic activities [3]. Explosive movements such as take-off, landing, sprinting, and rapid directional changes require powerful and rapid contractions of the gastrocnemius and soleus muscles [4] which can generate forces approaching 90% of their maximal capacity. These forces are transmitted directly through the Achilles tendon, resulting in substantial mechanical loading [5].

During athletic movement, particularly in the initial phase of ground contact, the ankle transitions into dorsiflexion while the Achilles tendon functions as a biological spring. Under eccentric contraction of the plantar-flexor muscles, the tendon elongates and stores elastic energy [6]. As dorsiflexion progresses beyond approximately 15°, muscle contraction shifts from eccentric to concentric [7]. At this point, the Achilles tendon must transition from elongation to shortening and rapidly increase its stiffness to effectively transmit concentric muscle force to the ankle joint. These abrupt functional transitions—from lengthening to shortening at specific ankle positions—expose the tendon to elevated mechanical stresses. These stresses are generated both by increased passive tension associated with gastrocnemius lengthening, and by near-maximal plantar-flexor muscle contraction, which may produce momentary forces exceeding the mechanical capacity of the tendon, thereby increasing the risk of partial or complete failure [8,9].

These high-risk transitions occur in two common athletic scenarios. First, injuries may arise when the foot is forcibly dorsiflexed while the calf musculature is actively contracting, such as during landing from a jump in basketball or volleyball.

Second, explosive push-off movements, including sprinting, jumping, and forward lunging (e.g., in soccer or basketball), generate extremely high plantarflexion forces that further increase tendon loading [10,11].

Knee joint position is an additional determinant of Achilles tendon loading. Most ruptures occur with the knee in full extension or close to full extension [12], when passive tension in the gastrocnemius is maximal. When combined with active soleus contraction, this produces the highest cumulative load on the tendon. As the knee flexes to deeper angles, and the ankle joint moves to deeper dorsiflexion, as occurs during landing, the contribution of the passive force of the gastrocnemius decreases, resulting in a reduction in overall Achilles tendon tension [12,13,14,15].

Despite the well-documented biomechanical risk factors and the high incidence of Achilles tendon injuries, effective strategies to dynamically unload the tendon during functional movement remain limited. The most commonly used intervention—heel elevation—reduces dorsiflexion and, consequently, decreases tendon strain. However, this approach offers only partial unloading, it alters gait biomechanics, and it does not mitigate the rapid lengthening-to-shortening transitions that characterize high-risk athletic movements [15,16].

Due to the specific biomechanical position of the ankle and the high angular velocities involved during explosive movements, currently available Achilles tendon unloading strategies—such as static bracing, heel-elevation insoles and taping—have demonstrated beneficial unloading effects only in cases of chronic tendon pain or tendinopathy. These approaches provide only a limited reduction in passive Achilles tendon loading during walking or running, without specifically targeting the initial dorsiflexion phase at around 15°, and without influencing the transition from eccentric to concentric muscle contraction, phases in which the Achilles tendon is at the highest risk of traumatic injury [17,18]. In light of these limitations, we propose a novel dynamic ankle brace designed to selectively unload the Achilles tendon when in its most vulnerable biomechanical state—specifically, at initial ankle dorsiflexion—while minimizing interference at other ankle joint positions.

To evaluate both concentric and eccentric unloading effects, we conducted a series of commonly used plantar-flexor muscle–tendon unit strength tests across different ankle and knee joint positions. Each test was selected to replicate distinct phases of explosive activities, including landing, push-off, and acceleration, thereby enabling a comprehensive assessment of the brace’s functional unloading performance under sport-relevant conditions.

## 2. Materials and Methods

### 2.1. Study Design: Controlled Laboratory Study

A total of 28 healthy young male basketball players took part in this study (age = 18.2 ± 0.6 yrs; height M = 1.82 ± 0.1 m; body mass index [BMI] M = 74 ± 3 kg).

The following inclusion criteria were applied: the athlete had no prior Achillies or plantar-flexor muscle injury or pain, had not undergone surgery on his lower extremities, and had no neurological/musculoskeletal condition that might hinder his ability to perform the physical tasks required in this study.

The study was approved by the Ethics Committee at the authors’ affiliated institution (reference number 12-010126, 1 January 2026). All participants submitted a signed consent form. In cases where participants were younger than 18 yrs, one parent also submitted their informed written consent.

### 2.2. Procedure

**Ankle range of motion measurements:** Prior to performing each heel-rise test, ankle range of motion was measured. Participants were instructed to wear shorts and to remove footwear, to allow clear visualization of anatomical landmarks. Prior to testing, each participant performed a standardized warm-up consisting of 5 min of comfortable walking. Reflective markers were placed on the tested limb at the following anatomical landmarks: lateral malleolus, fibular head, and fifth-metatarsal head. Marker placement was performed by the same examiner for all participants to ensure consistency. The subject stood barefoot on one leg and was instructed to raise the heel as high as possible and then slowly to lower the heel to the maximal point according to the type of test specification. The subjects performed the exercise three times. Every trial was recorded using a video camera. Ankle range of motion (ROM) in dorsiflexion and plantarflexion was defined as the maximal sagittal-plane angle between the shank segment and the foot segment, as determined by an experienced physiotherapist [19].

### 2.3. Tests and Tools

**Heel-rise tests:** After ankle ROM measurements were obtained, the subjects were randomly divided into two groups. One group performed concentric plantar-flexor maximal-strength tests (n = 14), and the second group (n = 14) performed maximal-velocity heel-drop eccentric exercise.

**The concentric** group performed the tests on two separate days. The test included testing the maximal strength of the plantar flexor of the dominant leg in three different positions, each simulating a different phase in athletes’ explosive activity. The testing order was randomized by the team coach, who was blinded to the study hypothesis involving one control group and one group with the dynamic brace.

1. *Full ankle range of dorsiflexion, knee flexed (FKF)*: to simulate athletic activity that includes the end stage of deceleration. To perform this test, the subject stood barefoot on one leg on a stair with the heel outside of the stair and not supported. The ankle was positioned at maximal dorsiflexion, and the knee was flexed at 30 degrees of flexion. The subject was then instructed to keep the knee immobile while moving the ankle joint from full dorsiflexion to full plantar flexion [20].

2. *Full-range dorsiflexion, knee extended* (FKE): to simulate injury mechanisms. This involved maximal dorsiflexion, with the knee extended. The subject stood barefoot on one leg on a stair with the heel was outside of the stair and not supported. The ankle was positioned at maximal dorsiflexion, and the knee was extended. The subject was then instructed to keep the knee extended, without movement, while moving the ankle joint from full dorsiflexion to full plantar flexion [21].

*Ankle natural position, knee extended* (NKE): to simulate the end phase of push-off. The subject stood on one leg on the floor. The ankle was positioned in the natural position, and the knee was extended. The subject was instructed to perform a maximal heel rise from full foot contact to maximal rise [22].

During all tests subjects were allowed to use one hand as support to maintain their balance. In all tests every heel rise was counted. Subjects were instructed to perform with maximal effort, and to stop when they felt fatigue or muscle pain. All tests were supervised by an experienced sports physiotherapist who ensured that each test was performed throughout the full target range of motion and that all subjects maintained an accurate ankle and knee position.

**Maximal-velocity eccentric heel-drop test**

Maximal-velocity eccentric full-range dorsiflexion, knee extended (EFKE): this test simulated injury mechanisms during forced dorsiflexion. The subject stood barefoot on one leg on a stair, with the heel outside of the stair and not supported. The subject was instructed to slowly raise the heel to maximal plantar flexion and then perform maximal dorsiflexion as fast as they could. Each subject repeated the trial 3 times [23]. To calculate motion duration, every attempt was recorded on 2D video, and the times taken between full plantar flexion and maximal dorsiflexion were analyzed by an experienced physiotherapist. The mean times of three attempts were then compared.

**The novel Achilles brace**

**Figure 1:**
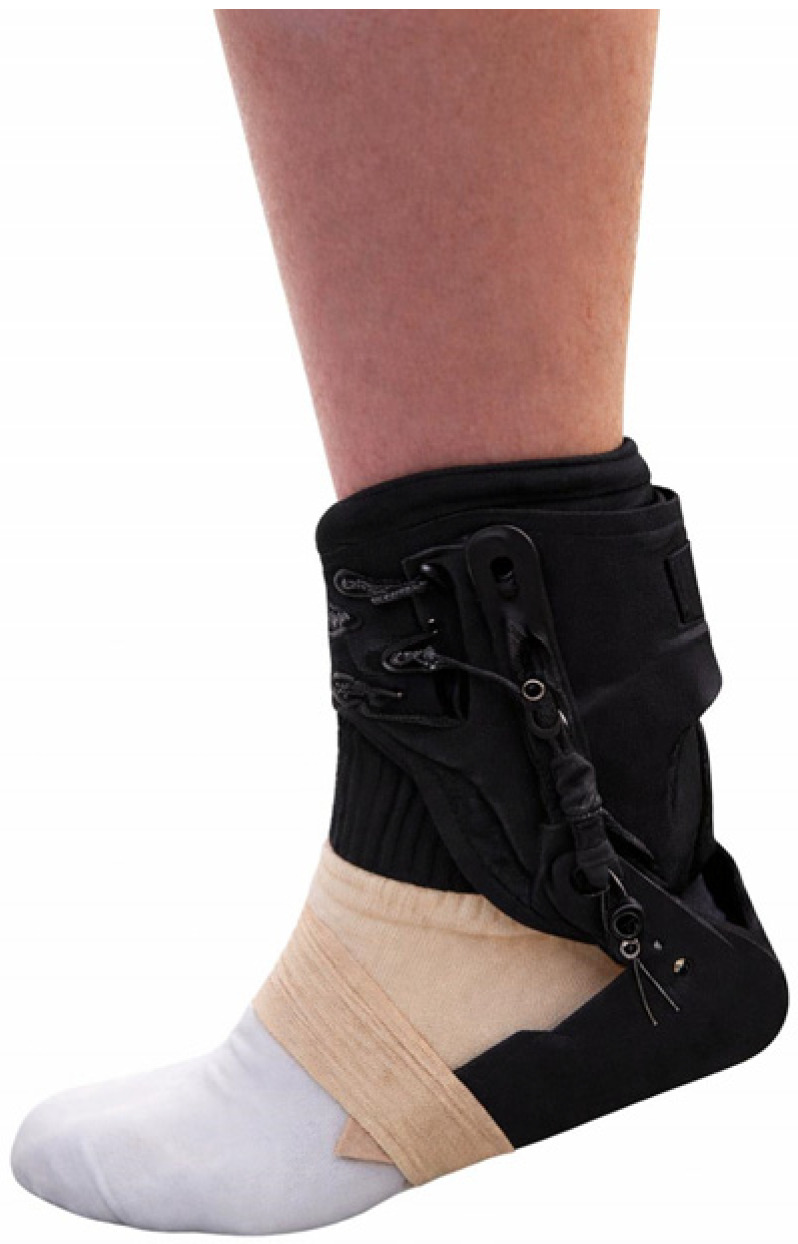
Dynamic heel brace used in this experiment.

The brace is shown in Figure 1. The brace design incorporates an adaptive mechanism that utilizes ankle dorsiflexion motion to generate spring tension, producing an additional plantar-flexor moment only in a high-risk Achilles position, without interfering with natural ankle motion. Its consists of a hinge brace with an axis at the level of the malleolus tuberosity. It has an upper attachment above the ankle joint and a lower attachment below the heel, this being attached to the heel by an upper band. The brace includes two springs, with one passing each axis. The springs are attached to the brace in such a way that, when under tension, they generate a pulling force at the ankle joint only in the plantarflexion direction, thereby assisting plantar-flexor motion and unloading the muscle tendon plantar-flexor unit.

Each spring produces a 20 N force at elongation of 1 cm. The brace is designed so that when the ankle is in the natural position no tension is applied by the spring. As users perform dorsiflexion, the spring is gradually tensioned, producing maximal tension at 15 degrees dorsiflexion. When dorsiflexion increases further, the tension in the spring reduces gradually, returning to zero at around 25 degrees and remaining at zero as dorsiflexion motion continues. There is no tension in the springs during plantar flexion motion.

The brace was fitted to each participant by an experienced physiotherapist. After donning the brace, each participant performed several ankle movements. Once comfortable and stable use was ensured, and there were no subjective reports of movement interference, testing proceeded.

### 2.4. Statistical Analysis

Parameters were compared. For concentric tests, these were ankle ROM and the maximal numbers of repetitions in the brace and control conditions. For the eccentric test, duration times were compared to ensure the robustness of the statistical evaluation, with descriptive statistical analyses and paired *t*-tests being conducted. Cohen’s *d* effect sizes were calculated to assess the magnitude of within-subject differences, and these were interpreted as follows: *d* < 0.20 = trivial, 0.21–0.50 = small, 0.51–0.80 = moderate, 0.81–1.10 = large, and *d* > 1.10 = very large. Statistical significance was set at *p* < 0.05 for all tests. An a priori power level of 0.80 (β = 0.20) was adopted for the study design. The statistical analyses were performed in accordance with the study design, and the findings were interpreted with appropriate caution. Data were analyzed using SPSS (IBM Corp., Armonk, NY, USA, Version 26).

## 3. Results

### 3.1. Range of Motion

As shown in Figure 1, no significant differences in ankle range of motion were observed between the brace and control conditions across all tests. As expected, significant differences in the maximal dorsiflexion range of motion were found between test conditions. The greatest dorsiflexion was observed in the FKF condition, at 34 ± 2.1°, a value significantly higher than in both the FKE condition (23 ± 1.0°) and the MVEKE condition (22 ± 2.0°, *p* < 0.05). The FKF value was also higher than that obtained for controlled dorsiflexion in the NKE test (1 ± 0.5°; *p* < 0.05). No significant differences in maximal plantarflexion range of motion were observed among the test conditions, with the following values being obtained: FKF, 43 ± 1.1°; FKE, 42 ± 1.8°; NKE, 44 ± 0.8°; MVEKE, 44 ± 2.0°.

In test results, negative values for ankle joint angles represent dorsiflexion motion, whereas positive values represent plantar flexion motion. The following abbreviations are used: PKF—full dorsiflexion, knee in flexion; FKE—full dorsiflexion, knee in extension; MVEKE—maximal velocity, eccentric, knee extended; NKE—ankle in natural position, knee extended. Blue—brace; orange—control.

### 3.2. Heel-Rise Test

**Figure 2:**
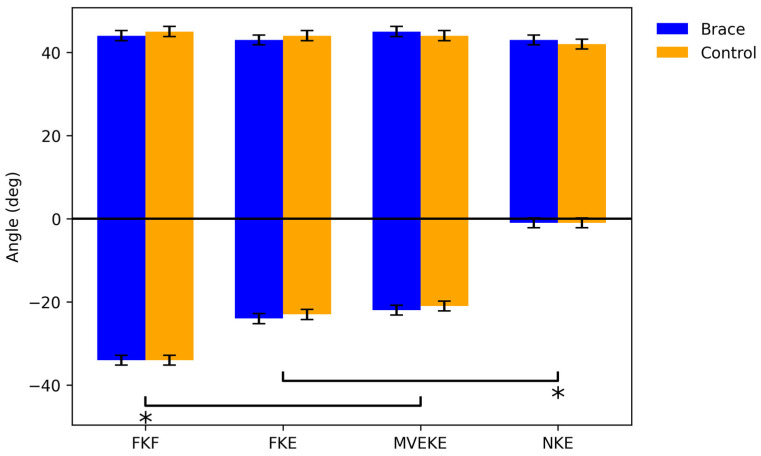
Ankle range of motion. * *p* < 0.05.

In the control condition, a significant difference in the maximal number of repetitions was observed between the FKF and FEK conditions (25 ± 1.3 vs. 21 ± 1.3 repetitions, respectively; *p* < 0.05). No significant differences were found between these tests and to the NKE condition (24 ± 1.3 repetitions) (Figure 2).

**Figure 3:**
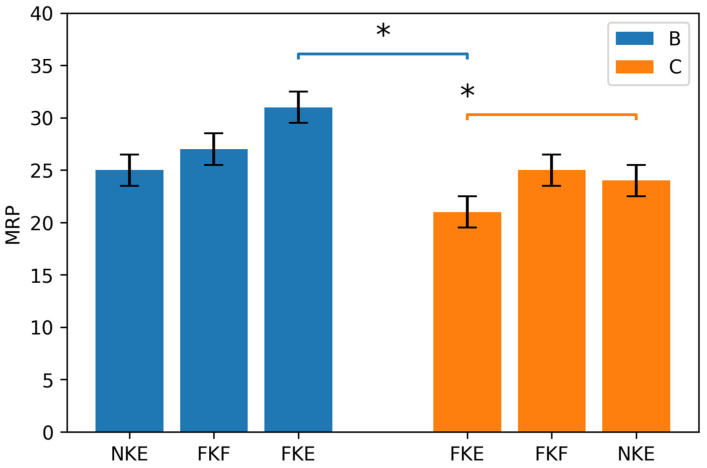
Maximal repetitions of heel-rise tests. * *p* < 0.05. FKF—full dorsiflexion with the knee flexed; FKE—full dorsiflexion with the knee extended; NKE—ankle in neutral position, with the knee extended. Blue—brace; orange—control.

When control and brace conditions were compared (Figure 3), it was found that wearing the brace resulted in a significant increase in performance only in the FKE condition (31 ± 1.3 repetitions), compared with its control condition (21 ± 1.3 repetitions; *p* < 0.05). No significant differences were observed between the FKE brace condition and the other brace conditions (FKF: 27 ± 1.3 repetitions; NKE: 25 ± 1.3 repetitions).

### 3.3. Maximal-Velocity Eccentric Ankle-Drop Test

**Figure 4:**
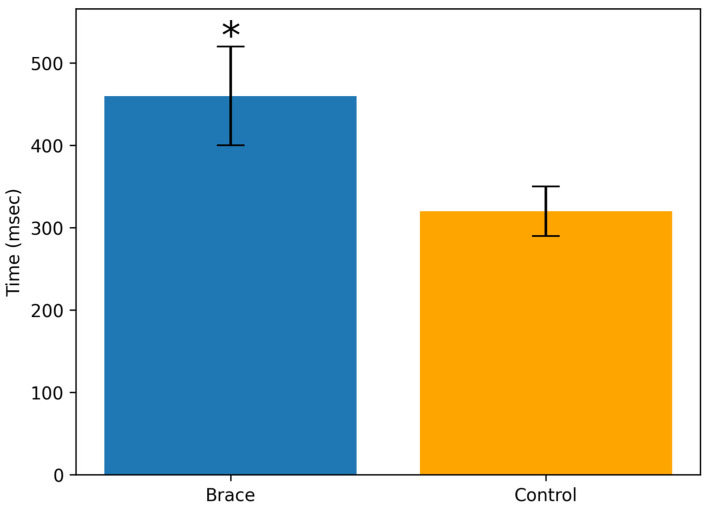
Maximal-velocity heel-drop test. * *p* < 0.05.

Wearing the brace resulted in a significantly longer transition time (Figure 4) from full plantarflexion to full dorsiflexion compared with the control condition (460 ± 60 msec vs. 320 ± 30 msec, respectively; *p* < 0.001).

## 4. Discussion

In the present study, we demonstrated that the dynamic Achilles brace produces several significant biomechanical effects during controlled effort in healthy subjects. First, it improves the concentric function of the plantar-flexor muscle–tendon unit at the lower dorsiflexion angles associated with high injury risk, such as those exhibited during vertical take-off. This was reflected in the significant increase in the number of heel-rise repetitions performed with the knee extended. Improving the muscle–tendon concentric function may produce a clinical benefit by unloading the tendon unit and providing protection during the concentric phase of near-maximal muscle contraction in explosive efforts [12,13]. Second, although the brace incorporates a spring mechanism, the short duration of its spring engagement did not improve concentric plantar-flexor performance during athletic tasks that typically required larger dorsiflexion ranges with the knee flexed. This finding indicates that the brace does not provide a performance advantage, and therefore may have potential for continuous use among athletes. Third, the brace improved eccentric plantar-flexor function, as indicated by a reduction in maximal ankle dorsiflexion velocity during the heel-drop test. This suggests improved resistance to eccentric loading, as occurs during landing or forced dorsiflexion, and which has been identified as a primary mechanism associated with Achilles tendon rupture in basketball [11]. Finally, unlike other Achilles tendon unloading methods, brace usage did not limit the natural range of ankle motion which is essential for athletic performance.

These findings may have important clinical implications, given the devastating consequences of an Achilles tendon rupture for an athlete’s career. This injury typically requires surgery and prolonged rehabilitation [1]. Previous research among NBA players has shown that only approximately 72% return to play, and also that, on average, it takes three years to regain pre-injury performance levels [23]. Currently, there is no proven intervention that clearly reduces the risk of Achilles tendon rupture. This is likely due to the combined effects of joint position and explosive loading at the time of injury. The mechanism involves near-maximal plantar-flexor contraction together with high-velocity ankle motion of approximately 300–330°/s [24].

Existing approaches such as heel-lifts, insoles [16,17], taping, and bracing [18] have been shown to moderately reduce Achilles tendon load by limiting dorsiflexion, with the largest effects being achieved at end-range dorsiflexion. However, the overall mechanical unloading achieved is only about 8–10% [17]. Contrarily, limiting ankle dorsiflexion may increase injury risk by altering lower-extremity mechanics; this has been associated with patellar tendinopathy and other musculoskeletal injuries [25]. Importantly, prolonged restriction of ankle dorsiflexion may alter tendon function and potentially increase the risk of Achilles tendinopathy [26]. In contrast, the dynamic brace evaluated in the present study is specifically designed to improve plantar-flexor mechanical function during early dorsiflexion. The spring tension begins at approximately 5°, peaks at 15°, and returns to zero by 25°, a plantar-flexion-directed assistive force being applied. This is relevant, because biomechanical and video-analysis studies of elite athletes [10,11] have shown that peak Achilles loading occurs when the gastrocnemius is at its maximal length, with the knee extended and the ankle in early dorsiflexion of around 15°, during the transition from eccentric to concentric loading in vertical jumping or during forced dorsiflexion [12,13,27].

Our control findings are consistent with previous research [15,20]. When heel-rise tests are performed on a flat surface, knee position does not influence maximal plantar-flexor performance. However, when performed on an incline, or with the heel over the edge as in the present study, knee flexion increases the number of repetitions due to greater soleus contribution. Furthermore, testing with an extended knee at close-to-maximal dorsiflexion reduces plantar-flexor performance compared with testing on a flat surface, likely due to increased gastrocnemius length [12,14]. In the present study, the combination of heel-over-the-edge testing and knee extension significantly reduced plantar-flexor performance, supporting the concept that this position represents a high-risk configuration: high passive tendon load [13], combined with reduced generation of gastrocnemius force. This also explains why the brace improved plantar-flexor function only under this condition. The spring tension at approximately 24° dorsiflexion matched the ankle angle achieved during testing, and the shorter ankle motion amplitude allowed longer spring engagement compared with the knee-flexed condition, in which dorsiflexion reached 35° and spring engagement occupied a smaller proportion of the total movement. No effect was seen during natural standing, when the springs were slack.

Importantly, the brace did not alter muscle performance during typical athletic movements. This suggests that chronic use is unlikely to modify tendon mechanical properties or neuromuscular performance, and therefore should not interfere with athletic function [28,29].

Eccentric testing further demonstrated that the brace significantly slowed ankle dorsiflexion velocity with the knee extended, simulating forced dorsiflexion injury [15] mechanisms. Given the strong association between tendon-lengthening velocity [4], tendon load, and tissue damage [30,31], these results imply that brace use may increase resistance to sudden high-velocity eccentric loading.

In addition, both concentric and eccentric tests indicated approximately a 30% improvement in plantar-flexor muscle–tendon function, compared with the ~10% reduction in tendon loading reported for heel-lifts and insoles [18,19,20]. This improvement was achieved using relatively low spring tension (20 N per spring). This was selected to preserve natural movement patterns, consistent with our finding that range of motion was not restricted. Future designs may allow spring-force adjustment according to clinical needs, potentially increasing tendon off-loading during rehabilitation following Achilles tendon rupture [29].

### Limitations and Future Research

This study examined only controlled strength-based tasks rather than explosive movements that more closely simulate injury mechanisms, primarily for safety reasons. In addition, all participants were healthy; individuals with prior Achilles rupture or tendinopathy may demonstrate different tendon mechanical responses. Furthermore, tendon loading was not directly measured, and no data were collected regarding ground reaction forces or ankle joint kinematics.

## 5. Conclusions

In summary, our findings suggest that the dynamic Achilles brace described here may improve both concentric and eccentric plantar-flexor muscle–tendon function at initial dorsiflexion angles associated with high risk of Achilles injury, without altering muscle performance. These findings indicate the potential of the brace to mechanically unload the tendon, and thus contribute to prevention of Achilles injuries. Future studies should further investigate the effects of the brace during explosive sport-specific maneuvers.

## Data Availability

The data presented in this study are available on request from the corresponding authors (R.G. and J.C.G.). The data are not publicly available due to ethical and privacy restrictions.
